# Watching or Listening: How Visual and Verbal Information Contribute to Learning a Complex Dance Phrase

**DOI:** 10.3389/fpsyg.2018.02371

**Published:** 2018-11-30

**Authors:** Bettina E. Bläsing, Jenny Coogan, José Biondi, Thomas Schack

**Affiliations:** ^1^Neurocognition and Action Research Group & Center of Excellence Cognitive Interaction Technology, Bielefeld University, Bielefeld, Germany; ^2^Department of Music and Movement in Rehabilitation and Therapy, Faculty of Rehabilitation Science, Technical University Dortmund, Dortmund, Germany; ^3^Palucca Hochschule für Tanz Dresden, Dresden, Germany

**Keywords:** motor learning, observation, verbal instruction, recall, performance, dance

## Abstract

While learning from observation is generally regarded as major learning mode for motor actions, evidence from dance practice suggests that learning dance movement through verbal instruction might provide a promising way to support dancers' individual interpretation of and identification with the movement material. In this multidisciplinary project, we conducted a study on the learning of dance movement through two modalities, observation of a human model in a video clip and listening to the audio-recording of a verbal movement instruction. Eighteen second year dance students learned two dance phrases, one from observation and one from verbal instruction, and were video-recorded performing the learned material. In a second learning step, they were presented the complementary information from the other modality, and their performance was recorded again. A third recording was carried out in a retention test 10 days after learning. Completeness scores representing the recall of the dance phrases, expert ratings addressing the performance quality and questionnaires reflecting the participants' personal impressions were used to evaluate and compare the performance at different stages of the learning process. Results show that learning from observation resulted in better learning outcomes in terms of both recall and approximation of the model phrase, whereas individual interpretation of the learned movement material was rated equally good after initially verbal and initially visual learning. According to the questionnaires, most participants preferred learning initially from observation and found it more familiar, which points toward an influence of learning habit caused by common training practice. The findings suggest that learning dance movement initially from observation is more beneficial than from verbal instruction, and add aspects with regards to multimodal movement learning with potential relevance for dance teaching and training.

## Introduction

Interdisciplinary projects linking dance and neurocognitive research have recently come to increasing awareness in artistic and scientific communities (see Sevdalis and Keller, 2011; Bläsing et al., 2012). Recently, the claim that such research should be carried out with equal contribution of and benefit for the different communities, by multidisciplinary teams involved at all stages, is been expressed with increasing emphasis (e.g., Jola, 2018). The project presented in this article represents an example of such research; it has been developed within an interdisciplinary network of scientists, scholars and artists (Dance engaging Science; The Forsythe Company|Motion Bank) and is motivated by dance-pedagogical questions on movement learning. The process of developing, planning and conducting this study has been monitored by the German society for dance medicine “tamed” (Tanzmedizin Deutschland e.V.), and the different stages of this process are presented and commented in a blog (www.blog.tanzmedizin.com), to provide a showcase for a multidisciplinary (German-speaking) audience. We expect that the outcomes contribute to our general understanding of movement learning in dance, and that they might yield potential implications for teaching and training in dance-related disciplines.

A decade after the discovery of the “mirror neurons” in the brains of macaque monkeys (Rizzolatti and Craighero, 2004) has given a new impetus for theoretical frameworks emphasizing the tight coupling of action and perception (Prinz, 1997; Hommel, 2015), scientists interested in related functions and systems in the human brain started to use video clips of dancers performing full-body movements as stimuli in brain imaging studies (Calvo-Merino et al., 2005, 2006; Cross et al., 2006). These influential studies showed that the activation of particular motor-related brain areas during the observation of human motor actions is modulated by the observer's own motor expertise (Calvo-Merino et al., 2005, 2006) and preference (Calvo-Merino et al., 2008). The interest in skilled motor action that resulted from such findings opened new roads for sport and exercise science to join forces with cognitive psychology and neuroscience in investigating action-perception coupling (Beilock, 2008; Moreau, 2015). An increasing number of studies addresses this and related topics in sports and dance contexts, as these are supposed to have a higher ecological relevance with regards to real-world scenarios (Jola et al., 2011). Beilock (2008) argues that the study of sport-specific scenarios has a high potential for advancing theories of cognitive neuroscience, in particular with regards to questions of motor control, motor learning, and expertise.

Motor actions from sports or dance are often referred to as naturally complex, in contrast to simple response actions (such as key presses) typically applied in experimental laboratory tasks. Despite the general use of the term “complex” with regards to motor actions or tasks, however, there seems to be no clear and reliable definition of that concept. Actions that are termed “complex,” as opposed to “simple,” often require specific training to be mastered even on a rather low level of performance (e.g., Meister et al., 2005; Cross et al., 2013), which makes them suitable for experimental learning tasks. Such actions typically involve the whole body (in sports or dance) or mainly the hands (in music or tool use), and consist of several independent elements that are either performed at the same time in a coordinated manner, or successively, as action sequences. In the case of action sequences, complexity often refers to the length of the sequence, the number of different components and the reproduceability of their order. The latter can be determined by a set of rules or an underlying “grammar,” which also contributes to the over-all complexity (e.g., Opacic et al., 2009). Hossner et al. (2015) suggest that complex tasks in sports have a modular architecture acquired by the athlete on the level of motor control, and that this architecture can be revealed as sub-goal-related micro-structure via a functional task analysis. According to Schack (2004), who also regards motor control as being constructed in a hierarchical manner, complex motor actions are based on mental representations in long-term memory that mediate between volition, or intention, and effect representations, with the latter being deterministic for simple movements. The general idea that motor control, motor learning and the performance of skilled actions are based on cognitive representations has been expressed by many authors, going back to Lotze (1852) and James (1981), as well as Bernstein (1947), who applied the idea of cognitive representations to his model of the construction of movement. Bernstein, 1935/1967 pointed out that movements should be understood as goal-directed acts and assigned a decisive role to the *model of the needed future* as organizing principle in movement control (Bernstein, 1947, 1967, 1975). Furthermore, he regarded the sophisticated control of particular movements characteristic of human skills is a consequence of action development, rather than its basis. Crucially, according to Bernstein's concept of dexterity developed in the 1940s, actions are primary, and simple movements and postures are consequences of the organism's activities rather than building blocks of action (Bernstein, 1996). Following this line of argument, Reed and Bril (1996) point out that the ability to construct, coordinate and modulate movements independently, regardless of the functional context, and to fractionate actions into postures and movements in a controlled way (as is done in dance), can be regarded as one of the most sophisticated human achievements.

Bernstein (1947, 1967, 1975) also emphasizes the role of sensory (re-afferent) feedback in this context, arguing that motor control requires a continuous processing of sensory feedback, as well as comparison with the coded effect. According to this view, visual and auditive feedback play a substantial role in the control of complex motor actions for controlling the multitude of degrees of freedom present in the motor system. Coordination is thus conceptualized as transforming the degrees of freedom of the movement system into targeted movement effects (see Bernstein, 1971). Such a transformation requires specific means, including cognitive ones (e.g., representations), and it requires a functional mediation between the different building blocks of the movement system. Bernstein (1947) presented the most comprehensive compilation of descriptive and experimental data on the functional mediation of the building blocks of the movement system available at that time. His model of the interplay between movement goals, representations, and perceptual feedback is composed of hierarchically organized interdependent levels, including a superordinate symbolic or conceptual level for the organization for complex movements. More recently, authors from different fields have emphasized the role of mental representations in the control and learning of motor actions (e.g., Glenberg, 1997, 2010). According to Steels (2003), mental representations primarily co-evolve together with the corresponding actions and thereby become vehicles for higher mental functions, such as thinking and planning. Nomikou et al. (2016) argue in favor of a continuous development of rich representations through and for action and interaction, suggesting that children develop rich representations from the beginning on, and propose that representations are continuously shaped and enriched throughout development by acting and interacting in the physical and social world.

While mental representations can be regarded as paramount for the learning, planning, adaptation and skilled performance of sophisticated movements as they are performed by athletes or dancers, the criteria applied to define the complexity of motor actions are still manifold. Wulf and Shea (2002) argue that no one continuum can be satisfactory for defining complexity with regards to task or action, but that a number of different context-dependent continua and their interactions, as well as the demands placed on the learner's capacity must be taken into account. The degree of complexity then depends on the choice of criteria applied, which might vary according to the context. Dance can be regarded as a domain in which actions require specific criteria for complexity. Tempel et al. (2015) refer to dance moves as definitely complex because they involve the whole body, have a hierarchical structure (i.e., they can be combined to higher level phrases), and they follow spatiotemporal rules (i.e., they have to be executed in a special order, corresponding to a given rhythm, and according to predefined spatial patterns). Furthermore, dancing requires practice and is embedded in a social and cultural context. An aspect that differentiates dance from most other action domains is that the absence of obvious external action goals is rather common. In contrast, dance movements often possess internal goals that are directly related to the movement itself, its trajectory, dynamics and expression. It has even been proposed that working memory might contain a kinaesthetic-spatial system in which body configurations act as goals, comparable to targets in external space (Smyth and Pendleton, 1990; Cortese Rossi-Arnaud and Rossi-Arnaud, 2010). Even though many motor actions performed in dance contexts also have external goals (depending on the choreography), these commonly do not supervene the movement-related goals that are typical for, and constitutive for, dance moves. Schachner and Carey (2013) refer to motor actions that do not possess external but movement-related goals as “dance-like,” even if these actions are not performed in a dance context, and state that dance-like actions are primarily characterized by their movement-based goals, whereas other “rational” actions have obvious external goals. Such dance-like actions, even though they do not easily comply to all criteria that have been identified for actions in general (such as an external goal), can be highly complex. With regards to learning, they are likely to depend more strongly on dynamic, movement-related representational formats than actions with external goals, and therefore to rely more strongly on internal simulation processes. Dance-like actions are definitely controlled by volition, require learning and practice, and can have a complex hierarchical structure, but the actual action goal is often hard to recognize for the naive observer, as it is predominantly related to movement parameters. Such actions can hardly be learned by emulation, but rather have to be acquired by imitation (i.e., copying results vs. copying actions, see e.g., Tennie et al., 2006), involving the direct route that is based on motor resonance rather than understanding of action goals and action semantics (Gonzalez Rothi et al., 1991; Rumiati et al., 2005).

Movement learning in dance therefore represents a specific type of motor learning that is characterized by a strong engagement of motor simulation processes, as well as by cognitive processes and strategies that depend on skill level. Novel movement material is typically taught in a multimodal manner, based on visual observation of a human demonstrator, supported by language, gesture, and body language providing kinematic, artistic, expressive and spatiotemporal cues, as well as the dancer's own advanced motor and imagery skills (e.g., Stevens and McKechnie, 2005; Stevens, 2017). It has been shown that the use of language (in terms of verbal cues) can facilitate or enhance motor learning by guiding attention toward relevant features of the movement and making these aspects explicit (see Wulf and Prinz, 2001). In dance and sports training, observational learning from a visual model is often supported by verbal cue-giving. Evidence from practice suggests that explicit verbal instructions and movement descriptions play an important supportive role in movement learning by providing conceptual clarity with regards to kinematic and spatiotemporal aspects and thereby fostering the understanding, simulation and performance of movement phrases. The role of language in motor learning, and in particular in the learning of complex movement skills, has been discussed mainly with a focus on verbal feedback (e.g., Magill, 1993) and attentional focus (e.g., Wulf and Prinz, 2001; Al-Abood et al., 2002). In addition to action execution, simulation and observation, the use of language (e.g., verbal instructions, verbal cues for imagery, explanation of complex moves, verbal feedback and error correction) can facilitate or enhance motor learning by guiding attention toward relevant features of the movement and thus making them explicit (Landin, 1994; Wulf and Prinz, 2001), or by adding semantic content to support the creative adaptation or expressive quality. Examples of such verbal comments could be: “make sure that your arms are stretched out in direct opposition, let the hands pull away from each other to the sides until you feel the pull in your shoulder blades,” or “don't just walk toward the front, but imagine you were approaching a long missed friend you have just spotted in the back of the audience,” respectively. Verbal instruction or feedback, however, can also interfere with motor learning by putting too much pressure on certain aspects and distracting from the movement flow (Wulf and Weigelt, 1997). While evidence from practice clearly shows that verbal cue giving plays an important role in dance on many levels (Waterhouse et al., 2014), it seems that the full potential of the use of language in the context of learning complex motor actions has not been exploited by research in the field.

In most dance disciplines, a repertoire of movement elements is built up through training that can then be combined into increasingly long and complex combinations. In modern and contemporary dance, especially on an advanced level, the creation of choreography goes far beyond the aligning of predefined steps and moves elements—in this case, learning of a new dance phrase would only mean to learn by heart the new sequence in which the familiar elements have to be concatenated; this, obviously, is not the case. Even in classical ballet, in which an extensive canon of more than 430 clearly defined movement elements is trained systematically (Puttke, 2018), learning new dance sequences on a higher skill level is far more than just lining up these elements, or “moving from pose to pose”—it is rather the acquisition and practice of a holistic movement “gestalt” that is characterized by its special dynamics, spatial and temporal parameters, expression and semantic and emotional content, as much as the body postures involved. In fact, advanced dancers are often less concerned with the poses or postures than with the transitions between them, making the flow of the movement progress continuously even at node points that look like breaks or goal postures to the observer. This “flow of energy” with its spatial, temporal dynamic and expressive features is what has to be learnt together with the movement “as such” when learning a dance phrase. In particularly in modern and contemporary dance, the composition of choreographed movement is less strictly bound to a predefined movement repertoire, but choreographers and dancers strive to explore and create novel ways of expressing themselves through the body. Modern dance of course has its own movement repertoire that it builds upon, but the emphasis is particularly strong on the flow of energy that characterizes the movement, making it novel, expressive, and special. Many choreographers therefore do not expect the dancers to simply reproduce movement phrases in adequate form, but to develop movement material on their own, in accordance with a given idea, description or instruction, aiming at a personal expression and special artistic quality of the developed movement material. In contemporary dance training, in particular, dancers are expected not only to reproduce movement material, but also to shape and develop movement material on their own, to achieve a more personal expression and higher artistic quality. Dance pedagogues educating future professional dancers emphasize the importance of their students achieving skills that enable them to interpret it with their own artistic quality, thus claiming ownership of the novel movement material rather than reproducing it. An assumption that had evolved in the context of dance training practice at the Palucca Hochschule für Tanz Dresden was that even though observational learning of dance movement had proved to lead to the best results in terms of correct movement reproduction, the presence of the visual model would at the same time interfere with the dancers development of movement ownership that would become visible in the expression of the performed movement as creative transformation of the learned movement.

To elucidate the roles of different modes of learning in dance in this context, we conducted a study with a group of second year dance students in which we compared the respective benefits of observational learning from a human model and learning from verbal movement description. Learning success was evaluated in terms of movement recall, performance of the learned material and personal preference. The dance students learned two comparable movement phrases via two different modes that were applied exclusively and in real-time: observation of a human model displayed in a video clip, and listening to a spoken movement description presented as audio recording. In a second step, the complementary mode of presentation was added. Between and after both learning steps, the students' physical performance of the learned material was recorded on video. A third video recording was produced during a retention test applied 10–14 days after the learning session to evaluate long-term effects. We expected that the dance students would reproduce the learned material more precisely after observational learning (hypothesis 1), but that their individual interpretation and personal liking for the learned material might be better for the material first learned from verbal description, due to a stronger embodiment and identification with the movement (hypothesis 2). The latter was hypothesized on the basis of evidence from practice gained by the teachers, who argued that due to the superiority of vision over the other senses, the students' perception of the movement would be drawn away from their own and toward the demonstrator's motor system, which would result in more precise reproduction but less personal adaption of the movement material. In contrast, when learning from verbal description, the students would have to rely more strongly on their own motor system to re-create the movement, thus giving it a more personal note and experiencing a stronger feeling of engagement. Furthermore, we expected that performance after the second learning step would be improved compared to performance after the first learning step in both conditions, due to beneficial effects of a combination of different modes of presentation and an increased amount of practice (hypothesis 3).

## Methods

### Participants

Eighteen students (age: 18.39 ± 1.04 years, range 17–20 years; one ambidextrous; 11 female) from the BA Dance study program (early second year) at the Palucca Hochschule für Tanz Dresden took part in the study without compensation or course credit. According to the Edinburgh Handedness Inventory applied before the experiment, 17 participants were right-handed and one was ambidextrous. The group of participants included native speakers of German (9), Portuguese (5), English (2), French (2), and Italian (2); two participants were bi-lingual. English was the commonly used working language in class, and all participants who were not native speakers of English indicated that their knowledge of English was at least good. Five of the participants engaged in recreational sports activities other than dance-related, including swimming, basketball, volleyball, soccer, and table tennis. All participants reported having normal or corrected-to-normal vision, and were naive with regard to the purpose of the experiment. This study was carried out in accordance with the recommendations of the ethics committee of Bielefeld University. A prospective ethics approval was not required in agreement with the institutional institution's guidelines and national regulations. All subjects gave written informed consent in accordance with the Declaration of Helsinki.

### Material

**Figure 1:**
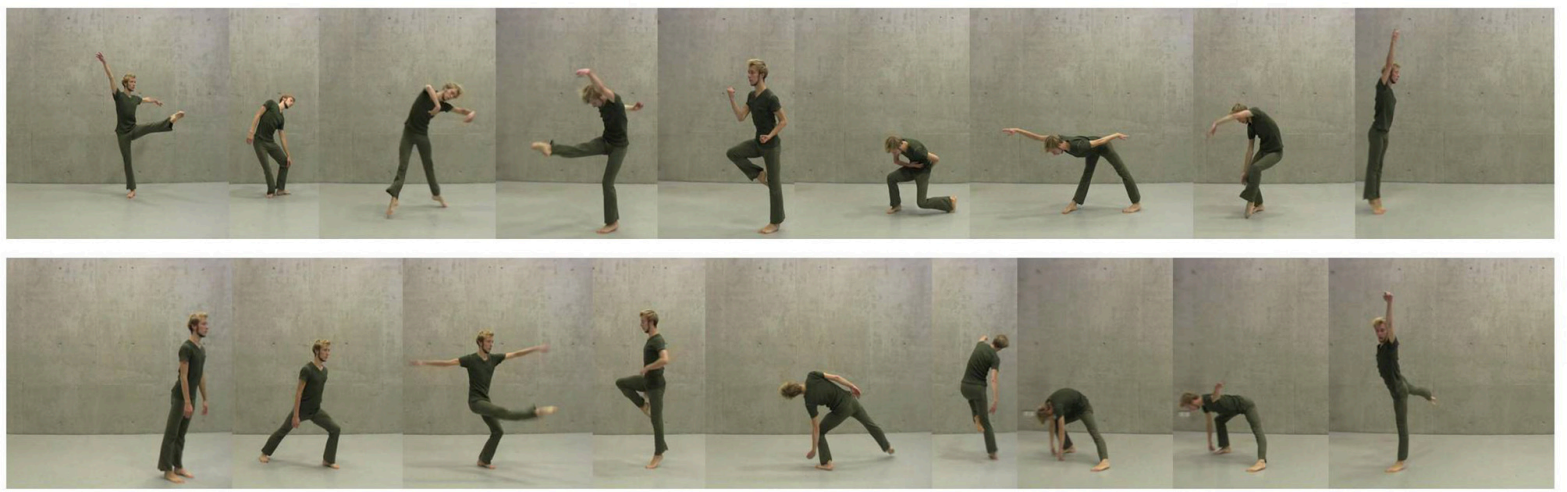
Images illustrating parts of the two dance phrases used in this study. Top panel: Phrase 1, choreographed by Jenny Coogan; the individual images correspond to movement elements 1–8 and 11 (end pose), as described in the Methods (completeness scores). Bottom panel: Phrase 2, choreographed by José Biondi; the individual images correspond to movement elements 1–6 and 8–10. Video clips of both dance phrases are provided as Supplementary Material. Dancer: Robin Jung (2013). Written informed consent was obtained from Robin Jung for the publication of this image in this article and the corresponding video clips in the Supplementary Material.

Two dance phrases were created as material for the learning task by two dance pedagogues teaching at the Palucca Hochschule für Tanz Dresden (co-authors Jenny Coogan and José Biondi). The dance phrases were choreographed in such a way that they were similar in length and complexity, each including a range of defined elements (such as turns, jumps, walks, changes of direction and height level). Both phrases were recorded on video in a dance studio at the Palucca Hochschule für Tanz Dresden, both danced by dancer and former Palucca student Robin Jung (Figure 1). In the study, the dance phrases were presented as video clips of 26 s each. For each of the two phrases, a verbal description was created by the choreographers that described full-body movements and movements of body parts in detail using every-day language (no particular dance-specific terms), including spatial and temporal cues. The following text represents an example from the movement description of Phrase 2: “Stand facing the left front diagonal of the room in parallel position. Feel the wind from the back that shifts your weight forward; let your upper body respond. Allow your body to move back and take the impulse again to move forward, allowing your weight to transfer from your heels to your toes. Once again shift back, this time falling onto your left leg, and follow with another step back, long and grounded, ending in a low lunge position, torso diagonal. Staying low, kick your right leg forward and your arms outwards to the sides as you twist your torso in opposition to the kick. Quickly bend your leg and arms into your center with a half turn to the right. Let the weight of your arms and center sink down on your left leg as your torso melts in a side-bend to the left and your leg extends sideways in opposition. Shift your weight onto the extended leg while your left arm describes a horizontal surface in front of you, reaching your torso over to the right side and bringing your left foot to the knee.”

The verbal descriptions were recorded as spoken audio files at the Palucca Hochschule für Tanz Dresden, the speaker was Alex Simkins. Durations of the audio files were 149 and 156 s for Phrases 1 and 2, respectively. Video clips and verbal movement descriptions as well as audio files of the recorded verbal descriptions are available as Supplementary Material.

### Procedure

The experimental learning sessions were carried out with all participants at the biomechanics lab at CITEC, Bielefeld University; during four consecutive days. Each participant was tested individually; the experimental session with each participant lasted ~1 h. After finishing the experimental session in the lab, the participant was asked to fill out a post-experimental questionnaire and was verbally interviewed. Before the experimental learning sessions started, each participant was assigned to one of four experimental groups (see Table 1: experimental design), with attention toward a balance with regards to gender and language background.

**Table 1:** Design of the experimental learning task.

**Learning session**		**Group 1A (*N* = 4)**	**Group 2A (*N* = 4)**		**Group 2B (*N* = 5)**	**Group 1B (*N* = 5)**
	**Consent, pre-experimental questionnaire**					
Step 1	Phrase 1	VERBAL 5x	VISUAL 5x	Phrase 2	VERBAL 5x	VISUAL 5x
Test		record < 3x	record < 3x		record < 3x	record < 3x
Step 2		VISUAL 2x	VERBAL 2x		VISUAL 2x	VERBAL 2x
Test		record < 3x	record < 3x		record < 3x	record < 3x
Step 1	Phrase 2	VISUAL 5x	VERBAL 5x	Phrase 1	VISUAL 5x	VERBAL 5x
Test		record < 3x	record < 3x		record < 3x	record < 3x
Step 2		VERBAL 2x	VISUAL 2x		VERBAL 2x	VISUAL 2x
Test		record < 3x	record < 3x		record < 3x	record < 3x
	**Post-experimental questionnaire, interview**					
Retention		*(N = 3)*	*(N = 4)*		*(N = 4)*	*(N = 2)*
Test	Phrases 1, 2	record 1x	record 1x	Phrases 1, 2	record 1x	record 1x
	**Retention questionnaire**					

*Dark gray, visual-first condition; Light gray, verbal-first condition*.

At the beginning of the individual experimental learning session, the participant entered the biomechanics lab and was introduced to the experimenters and the technical set-up. Both dance pedagogues were also present during the entire session. The participant was then equipped with 42 retro-reflective markers, as parts of the experimental session were recorded with a Vicon motion capture system (12 infra-red camera), in addition to the two video cameras positioned at different locations in the lab (in this article, only video-based results are presented; results of the motion-capture will be presented separately). Before the experimental session started, the participant was asked to fill out the necessary forms (e.g., consent) as well as a pre-experimental learning type questionnaire. The participant was then asked to enter the recording space in the middle of the lab and instructed how to use the space to allow for optimal visibility for the Vicon system and the video cameras. Subsequently, the participant was verbally given the learning task instructions (depending on the experimental design, see Table 1) by the main experimenter. The participant was instructed to learn two dance phrases of similar length and complexity, each through a combination of visual observation and verbal movement description. Depending on the group the participant had been assigned to, the individual participant's session either started with verbal or visual learning of either Phrase 1 or Phrase 2 (see Table 1). In the following, the learning of a particular dance phrase starting with visual learning (observation of the video clip) will be referred to as Visual-first condition, and the learning task starting with learning from verbal description (listening to the audio clip) will be referred to as Verbal-first condition.

For better comprehensibility, the procedure is described here for a participant assigned to group 1A, as example; this participant learned Phrase 1 in the Verbal-first condition and Phrase 2 in the Visual-first condition. This means that the participant first learned Phrase 1 from verbal description only (Step 1) with visual information added subsequently (Step 2). Then, the participant learned Phrase 2 from visual observation only (Step 1) with verbal information added subsequently (Step 2).

In the first learning step, an audio clip of the spoken verbal description of Phrase 1 was played five times consecutively. The participant was instructed to learn the movement sequence and was allowed to move or mark the movement as required while listening in order to support the learning process. After the fifth time listening to the audio file, the participant was allowed to try out the learnt movement phrase once; then s/he was recorded performing the learnt phrase. Up to three trials of the phrase were recorded and the best performance was marked, according to the participant's decision and preference. In the second learning step, the same dance phrase was presented as video clip twice, and the participant watched while being allowed to move or mark *ad libitum*. After the second watching, the participant was again allowed to try out and then was recorded again up to three times, as before. Two repetitions were chosen at this stage because no learning of new movement material was involved; instead, the novel information was supposed to be used only to compare and update the previously learned and practiced material. After a short break, the participant was presented Phrase 2 five times consecutively as video clip (Step 1), and the participant watched while moving as required. After trying out, the participant was recorded up to three times performing the new phrase. Then the verbal description of Phrase 2 was played twice while the participant listened and moved *ad libitum* (Step 2), and the phrase was recorded again, as before. After the markers were removed from the participant's body, s/he was debriefed and led to another room to fill out the post-experimental questionnaire and, finally, was interviewed about his or her personal impression of the experiment (the interviews were recorded for teaching-related purposes and are therefore not considered here).

### Retention test

Ten days after the last day of learning sessions (i.e., 10–13 days after the individual participants' learning sessions), a retention test was carried out at the Palucca Hochschule für Tanz Dresden. The retention test took ~10 min for each participant; all 13 participants were tested consecutively on the same day. Five out of the 18 participants were not present on that day due to illness or injury and therefore did not participate in the retention test. The remaining thirteen dance students (8 women) were called individually from the on-going training session to a free dance studio and were asked to dance the two dance phrases from the experimental learning session as completely and perfectly as possible. Each participant was allowed to practice for several minutes and then was instructed to dance the phrases in arbitrary order while being recorded with a video camera. The participants were not given any verbal or other assistance in reproducing the phrases, and they had not been informed in any way that there would be a retention test or that the movement material learned during the experimental session would be needed later on. After the recording, the participant filled out a post-retention questionnaire and was taken back to the dance class after being instructed not to convey any information to the other participants.

### Evaluation and analysis of the video material

From each participant, six video clips were used for the evaluation, three for each dance phrase. Each video clip was representative for the student's performance of the phrase at a particular recording time: after learning Step 1 (one modality), after learning Step 2 (two modalities), and after the retention test. If two or three trials had been recorded at a given recording time, the trial that the participant had indicated as the preferred one was chosen. For the evaluation, the six selected video clips of each participant were named assigned names that included the phrase and an abstract code (e.g., P1_xyz) that did not give away the recording time (Step 1, Step 2, Retention) or the learning condition (Visual, Verbal). In total, 98 video clips (4 × 18 = 72 from the experimental learning session, 2 × 13 = 26 from the retention test) were used for the evaluation.

### Completeness scores

All video clips were annotated for their completeness by two independent annotators, both advanced students of sport science who were experienced with analyzing human motor action from video material, who had learned each movement phrase in detail from both the video and the verbal description. As basis for the evaluation of completeness, each dance phrase was segmented into eleven sub-phrases or elements (note that the phrases had been choreographed to resemble each other in complexity, duration and structure). Both annotators independently watched all 98 video clips in randomized order and rated for each of the eleven movement elements if it was present and correctly executed (1.0 points), missing (0.0 points), or in between (e.g., half present/correct: 0.5 points; almost correct: 0.8). Each annotator produced a score between 0.0 and 11.0 for each video clip. This way, a minimum score of 0.0 would indicate that the phrase was not danced at all, or was not at all recognizable, whereas a maximum score of 11.0 would indicate that the phrase was performed in completeness and without error. The ratings of the two annotators were then averaged.

### Expert ratings

Two professional dance pedagogues teaching on the level of professional dance education comparable to the level of the participants who were not involved in the study otherwise and were naive toward the conditions and instructions and the experimental design were assigned as independent experts. Both experts to learned each movement phrase in detail from both the video and the verbal description and then independently watched and rated the 72 video clips from the experimental learning session in randomized order using a standard score sheet that contained a pre-defined list of criteria. The score sheet contained 15 questions (14 six-point Likert-type questions) assigned to two main categories, Approximation of the Model (AMo) and Individual Interpretation (IndI). Nine questions, or criteria, were addressed explicitly with regards to the approximation of the model phrase (AMo), namely:

A1.1 Clarity of movement initiation and pathway through the body

A1.2 Spatial orientation in external space (allocentric)

A1.3 Spatial orientation in external body-related space (egocentric)

A1.4 Temporal differentiation (proportion of the parts of the sequence in relation to one another)

A1.5 Connectedness, fluency of the movement

A1.6 Performance, over-all in relation to the model

A2.1 How much does Part 1 of the Phrase resemble the model?

A2.2 How much does Part 2 of the Phrase resemble the model?

A2.3 How much does Part 3 of the Phrase resemble the model?

The three parts addressed in questions A2.1-3 corresponded to elements 1–5, 6–7, and 8–11 (Phrase 1) and elements 1–4, 5–9, and 10–11 (Phrase 2) used as basis for the completeness scores, respectively.

Six criteria were addressed with regards to the Individual Interpretation (IndI), independent of the model phrase:

B1.1 Clarity of movement initiation and pathway through the body

B1.2 Spatial orientation in external body-related space (egocentric)

B1.3 Phrasing, temporal differentiation

B1.4 Connectedness, fluency of the movement

B1.5 Performance quality

B2 Did the phrase include the following elements?

Pause/suspension/successional movement/simultaneous movement

For each clip, the ratings of the two experts were averaged. Additionally, ratings for questions A1.1-6 (A1), for questions A2.1-3 (A2) and for questions B1.1-5 (B1) were averaged to achieve over-all ratings for each category.

### Questionnaires

Before the experiment, participants filled out a questionnaire to determine their learning type (e.g., Kirby et al., 1988). The questionnaire was shaped to mainly differentiate visual from verbal learners, it was based on the more extended Index of Learning Styles Questionnaire by Litzinger et al. (2007). Eight out of the 16 questions focused on this difference, the other eight questions were mainly added to make this purpose less obvious for the participants. Questions were phrased in the following way (example): “When I think about what I did yesterday, I am most likely to get: (a) words (b) a picture,” with one option always referring to the category “verbal” and the other one to the category “visual.” For the eight relevant questions, one point was added for the category the participant had chosen; if the participant had marked both answers, each category scored 0.5 points. Each participant was assigned to the category in which s/he had scored two or more points more than in the other category; if the difference was smaller than two points, the participant was defined as mixed-type learner.

After the experimental procedure and after the retention test, participants filled out questionnaires evaluating their impressions of the task and of their own performance. The post-experimental questionnaire included learning task specific questions for each condition (e.g., how confident did you feel when dancing the sequence after learning it from observation/from verbal instruction? How clear did you find the video demonstration/verbal instruction? How clear did you find the additional verbal/visual information?) and general questions (e.g., how competent do you consider yourself at learning from observation/from verbal instruction? How much do you enjoy learning from observation/from verbal instruction?). The retention questionnaire included only learning task specific questions for each condition (e.g., how difficult did you find this dance phrase? How difficult did you find it to remember the sequence? How much did you like this dance phrase? How confident did you feel when dancing the sequence?).

## Results

**Figure 2:**
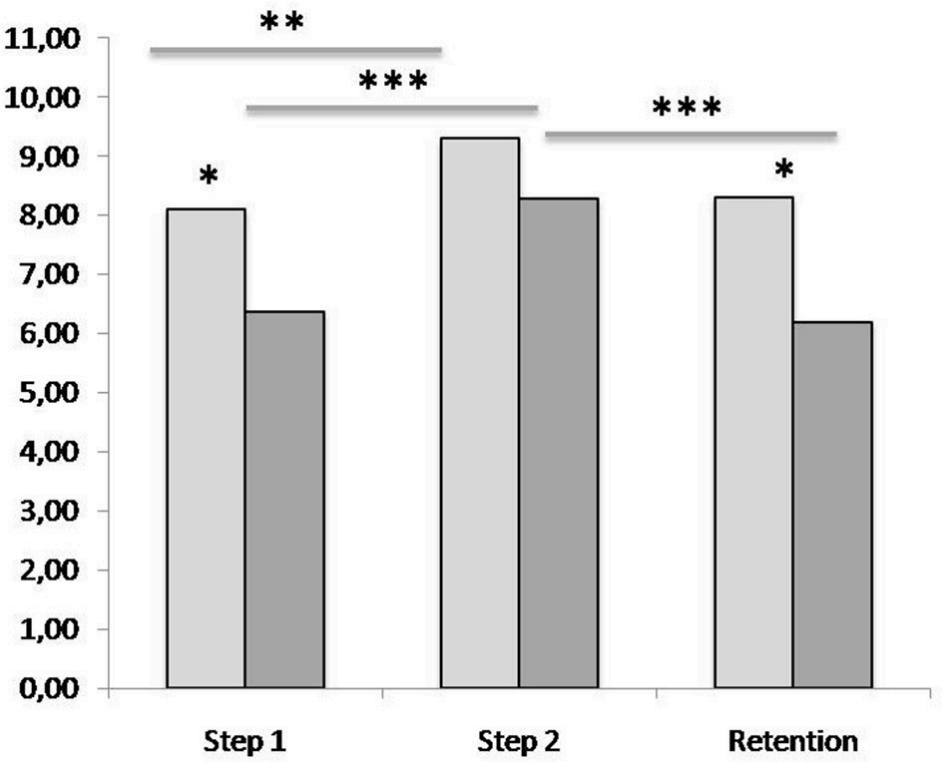
Completeness scores for video recordings of participants' performance of the two dance phrases learned in the experimental task at three occasions. Light gray: Visual-first condition; dark gray: Verbal-first condition. Step 1: participant's performance recorded after learning from either visual (Visual-first condition) or verbal (Verbal first condition) information (five repetitions). Step 2: participant's performance recorded after receiving the complementary (verbal or visual) information (two repetitions). Retention: participant's performance 10–13 days after the experimental learning session (unprepared test). Numbers on the *y*-axis refer to scores given by the two annotators (averaged) for the performance of 11 elements, or sub-phrases, of the dance phrases; for each element, each annotator could give a score between 0.0 (element missing) and 1.0 (element performed completely and without error), resulting in a maximum score of 11.0 for the entire phrase. Asterisks refer to significance levels of comparison of means: *p ≤ 0.05; **p ≤ 0.01; ***p ≤ 0.001.

Completeness scores given by the two annotators were highly correlated (Steps 1 and 2: *r* = 0.903, *p* < 0.001; Retention: *r* = 0.957, *p* < 0.001), therefore completeness scores of the two annotators were averaged for the further analysis. After confirming normal distribution of the data (Shapiro-Wilk test), a 2 × 3 ANOVA with factors CONDITION (Verbal-first, Visual-first) and TIME (Step 1, Step 2, Retention) revealed main effects of CONDITION [*F*_(1, 12)_ = 9.286; *p* = 0.010] and TIME [*F*_(1.16, 13.97)_ = 11.702; *p* = 0.003], but no interaction. A 2 × 3 ANOVA with factors PHRASE (Phrase 1, Phrase 2) and TIME (Step 1, Step 2, Retention) revealed a main effect of TIME [*F*_(1.16, 13.97)_ = 11.702; *p* = 0.003], but no effect of PHRASE and no interaction. Violation of the sphericity assumption resulted in a correction of the *p*-values and degrees of freedom according to Greenhouse-Geisser. As *post-hoc* comparison, paired *T*-tests were used to compare the averaged completeness scores between learning conditions (Visual-first, Verbal-first), learning steps (Step1, Step 2), and dance phrases (Phrase 1, Phrase 2). After the first learning step, completeness scores were better for the phrase learned in the Visual-first condition (mean completeness score: 8.10) than for the phrase learned in the Verbal-first condition [mean completeness score: 6.36; *t*_(17)_ = 2.905, *p* = 0.010], whereas no difference between the conditions was found after the second learning step [Visual-first: 9.29; Verbal-first: 8.27; *t*_(17)_ = 2.010, *p* = 0.061]. After the retention, completeness scores were again better for the phrase learned in the Visual-first condition (mean completeness score: 8.30) than for the phrase learned in the Verbal-first condition [mean completeness score: 6.19; *t*_(12)_ = 2.526, *p* = 0.027]. Within both learning conditions, completeness increased from Step 1 to Step 2 [Visual-first: *t*_(17)_ = −3.591, *p* = 0.002; Verbal-first: *t*_(17)_ = −5.191, *p* < 0.001]. Only in the Verbal-first condition, completeness dropped significantly from Step 2 to the retention [*t*_(12)_ = 6.832, *p* < 0.001]. Results for completeness scores are displayed in Figure 2.

**Figure 3:**
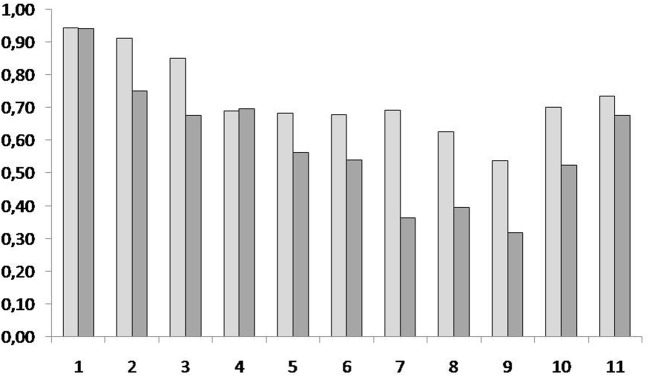
Completeness scores for video recordings of the participants' performance of the two dance phrases learned in the experimental task. Light gray: Visual-first condition; dark gray: Verbal-first condition. Numbers on the *x*-axis refer to the 11 elements, or sub-phrases, each dance phrase was divided into; numbers on the *y*-axis refer to scores given by the two annotators (averaged) for the performance of these 11 elements; for each element, each annotator could give a score between 0.0 (element missing) and 1.0 (element performed completely and without error).

Comparison of the completeness scores for the eleven individual elements of both phrases revealed higher scores for the first 3 and 2 elements in the Verbal-first condition and the Visual-first condition, respectively (see Figure 3; Table A, Supplementary Material), which points toward a primacy effect that was more pronounced in the Verbal-first than in the Visual-first condition.

### Expert ratings

**Figure 4:**
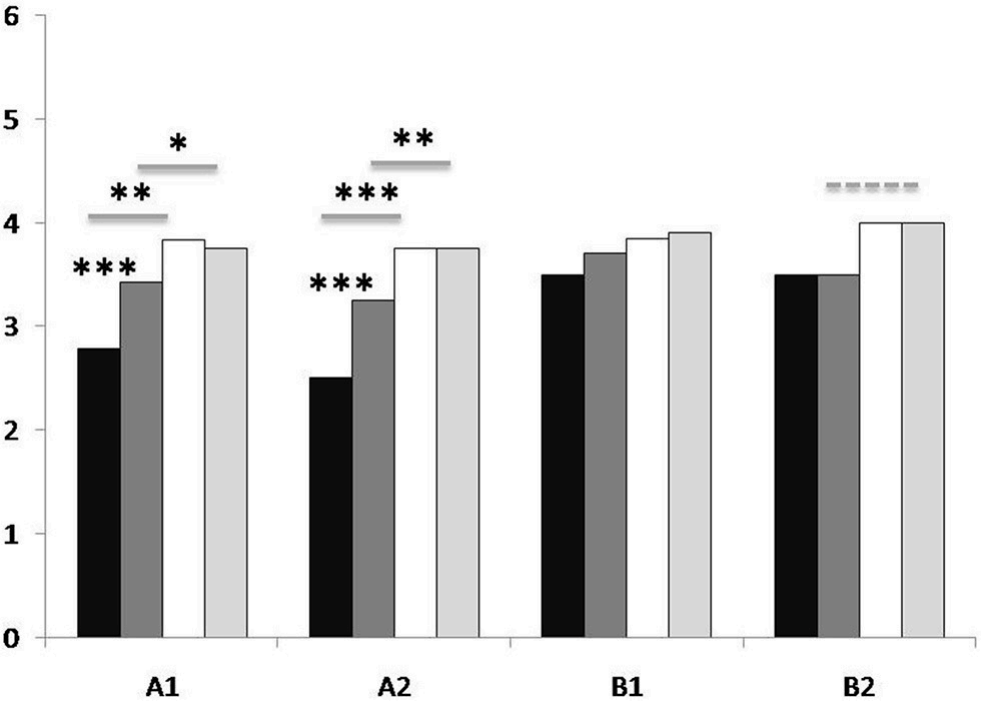
Expert ratings for participants' recorded performance according to the criteria Approximation of the model (AMo) and Individual Interpretation (IndI). Black: Verbal-first condition, Step 1; dark gray: Verbal-first condition, Step 2; white: Visual-first condition, Step 1; light gray: Visual-first condition, Step 2. Numbers on the y-axis refer to experts' ratings on a six-point Likert scale used to evaluate the performance of the dance phrases (note that for B2, 4 is the maximum value). Labels on the *x*-axis: A1 refers to AMo criteria A1.1-6: Clarity of movement initiation and pathway through the body; Spatial orientation in external space (allocentric); Spatial orientation in external body-related space (egocentric); Temporal differentiation (proportion of the parts of the sequence in relation to one another); Connectedness, fluency of the movement; Performance, over-all in relation to the model). A2 refers to AMo criteria A2.1-3: (“How much does Part 1/Part 2/Part 3 of the Phrase resemble the model?”). B1 refers to IndI criteria B1.1-5: Clarity of movement initiation and pathway through the body; Spatial orientation in external body-related space (egocentric); Phrasing, temporal differentiation; Connectedness, fluency of the movement; Performance quality. B2 refers to question B2: “Did the phrase include the following elements: pause/suspension/successional movement/simultaneous movement?” For each clip, the ratings of the two experts were averaged. Additionally, ratings for questions A1.1-6 (A1), for questions A2.1-3 (A2), and for questions B1.1-5 (B1) were averaged to achieve over-all ratings for each category. Asterisks refer to significance levels of comparison of means: *p ≤ 0.05; **p ≤ 0.01; ***p ≤ 0.001; dashed line: tendency (*p* = 0.052).

Ratings of the two experts were positively correlated for both main categories (AMo: *r* = 0.528; *p* < 0.001; IndI: *r* = 0.513; *p* < 0.001). Non-parametric tests (Wilcoxon signed-rank tast) were used to compare the averaged ratings for each individual question and categorial ratings A1, A2 and B1 between the four conditions (Visual-first Step 1, Visual-first Step 2, Verbal-first Step 1, Verbal-first Step 2). In the Verbal-first condition, all individual AMo ratings (questions A1.1-6 and A2.1.3) were higher after Step 2 than after Step 1 (see Table 2; Table B, Supplementary Material). In the Visual-first condition, no differences between the Step 1 and Step 2 were found. After Step 1 and after Step 2, AMo ratings for the Visual-first condition were generally better than for the Verbal-first condition (exception: Step 2 A1.6; tendencies for A1.1). Comparison of the ratings for the first, middle and last part of each dance phrase (questions A2.1-3) revealed that AMo ratings for the first part were better than for the middle part in all conditions (Visual-first Step 1: *p* = 0.007; Visual-first Step 2: *p* = 0.001; Verbal-first Step 1: *p* < 0.001; Verbal-first Step 2: *p* < 0.001) and better than the last part in three out of the four conditions (Visual-first Step 2: *p* = 0.011; Verbal-first Step 1: *p* = 0.001; Verbal-first Step 2: *p* = 0.006), whereas no difference was found between the ratings for the middle and last part (see Figure 4). Averaged AMo ratings for A1 and A2 are displayed in Figure 4.

**Table 2:** Medians of expert ratings.

	**Verbal-first, Step 1**	**Verbal-first, Step 2**	**Visual-first, Step 1**	**Visual-first, Step 2**
**AMo**				
A1.1	3	3.5	3.75	3.5
A1.2	2.5	3	3.5	4
A1.3	3	3.5	3.75	4
A1.4	2.5	3.5	3.5	4
A1.5	3	3.25	4	3.5
A1.6	3	3.5	3.5	3.5
A1	2.79	3.42	3.83	3.75
A2.1	3	3.75	4	4.5
A2.2	2	3	3.5	3.5
A2.3	2	3	3.75	3.75
A2	2.50	3.25	3.75	3.75
**IndI**				
B1.1	3.5	3.5	4	4
B1.2	4	4	4.25	4.5
B1.3	3.5	3.5	3.75	3.5
B1.4	3.5	3.75	4	3.5
B1.5	3.5	3.75	3.75	4
B1	3.50	3.70	3.85	3.90
B2	3.5	3.5	4	4

*AMo, approximation of the model; IndI, individual interpretation; cells with categorical ratings A1 (A1.1-6), A2 (A2.1-3), and B1 (B1.1-5) are marked in gray*.

IndI ratings did not differ between learning steps in either condition. After Step 1, IndI ratings for the Visual-first condition were better than for the Verbal-first condition for questions B1.2-4, but not for the average B1 rating. After Step 2, only tendencies were found for questions B1.2 and B2. Medians for all categories and results of all tests are displayed in Table 2. Results of the averaged IndI ratings (B1) are displayed in Figure 4.

### Questionnaires

Post-experimental and post-retention questionnaires were analyzed comparing participants' mean responses for the two learning conditions (Wilcoxon signed-rank) and correlating responses to each other and to completeness scores and expert ratings (Spearman's rho). According to the post-experimental questionnaires, the students perceived the information provided by the video clip as clearer than the information provided by the audio text, both in Step 1 (*Z* = −3.002, *p* = 0.003) and in Step 2 (Z = −2.547, *p* = 0.011). In the Visual-first condition, feeling confident dancing after Step 1 was negatively correlated to finding the additional verbal information in Step 2 clear (*r* = −0.556, *p* = 0.018) and useful (*r* = −0.577, *p* = 0.012), and finding the additional information ins Step 2 useful was positively correlated to finding it useful (*r* = 0.762, *p* < 0.001) and feeling confident dancing afterwards in Step 2 (*r* = 0.494, *p* = 0.037). In the Verbal-first condition, feeling confident dancing after Step 1 was positively correlated to finding the verbal instruction clear (*r* = 0.650, *p* = 0.003) and feeling confident dancing after Step 2 (*r* = 0.499, *p* = 0.035). In general (i.e., independent of the experimental task) participants enjoyed learning from observation better than learning from verbal instruction (*Z* = −2.084, *p* = 0.037), they considered learning from observation as more useful than learning from verbal instruction in dance (*Z* = −3.028, *p* = 0.002) and they were more familiar with learning dance movement from observation than from verbal instruction (*Z* = −3.458, *p* = 0.001). Enjoying learning was positively correlated to feeling competent for both learning modes (verbal: *r* = 0.737, *p* < 0.001; visual: *r* = 0.623, *p* = 0.006). Being familiar with learning from verbal instruction was positively correlated to feeling competent for it (*r* = 0.725, *p* = 0.001), enjoying it (*r* = 0.638, *p* = 0.004) and finding it useful (*r* = 0.464, *p* = 0.052).

The retention questionnaires revealed that in both conditions (marginal for the Verbal-first condition), liking a phrase was negatively correlated to finding it difficult to remember (Visual-first: *r* = −0.746, *p* = 0.003; Verbal-first: *r* = −0.550, *p* = 0.051). In the Verbal-first condition, liking a phrase was negatively correlated to finding it difficult to dance (*r* = −0.599, *p* = 0.030), and feeling confident dancing a phrase was negatively correlated to finding it difficult to remember (*r* = −0.871, *p* < 0.001) and difficult to dance (*r* = −0.612, *p* = 0.026). For both conditions, finding it difficult to remember a phrase was negatively correlated to the retention test completeness scores (Visual-first: *r* = −0.557, *p* = 0.029; Verbal-first: *r* = −0.621, *p* = 0.024). In the Verbal-first condition, feeling confident dancing a phrase was positively correlated to the retention test completeness scores (*r* = 0.628, *p* = 0.028).

### Learning type questionnaires

According to the learning type questionnaire, seven participants (four females) were assigned to the visual learners and six (two females) were assigned to the verbal learners; the remaining five participants were mixed-type learners. Completeness scores, expert ratings and questionnaire results of visual (*N* = 7) and verbal (*N* = 6) learners were compared, however, no significant differences between the learning-type groups were found.

## Discussion

In a study with 18 second year dance students, the participants' performance of two dance phrases learned under different conditions was compared. Each participant learned one phrase initially via listening to a verbal movement description (Verbal-first condition) and the other one via observation of a human model in a video clip (Visual-first condition). In a second learning step, the complementary modality of information was presented. In a retention test ~10 days after learning, students were asked unexpectedly to recall and perform both learned dance phrases. Completeness of the dance phrases performed by the participants was evaluated on the basis of video clips recorded at three points in time, as measures of learning success in terms of recall. The three recordings were produced after the first and the second learning step of the experimental learning task and at the retention test. Additionally, expert ratings for two main criteria, approximation of the model phrase (AMo) and individual interpretation (IndI), were used to evaluate the quality of the performance after the first and the second learning step from a dance-pedagogical perspective. After the experimental learning task and after the retention test, questionnaires were applied to evaluate the participants' personal impressions.

Completeness scores showed that recall was generally better after the second learning step than after the first (i.e., after learning from both modalities compared to only one modality). This finding can be interpreted as supporting the view that information from different modalities is beneficial for the learning of a motor task. In real world dance learning situations, movement is hardly ever learnt through one modality alone, but from visual observation of movement typically demonstrated by the teacher, complemented by verbal cue-giving and instruction and supported by the dance student's own motor action. An explanation for the superiority of combined learning modes compared to learning through one modality alone is provided by the perspective that during motor learning and practice, information from all sensory modalities is integrated and merged into rich action representations that are perceived as consistent and meaningful (Zacks et al., 2007; Barsalou, 2008; Nomikou et al., 2016). Such representations in long-term memory are thought to comprise declarative and non-declarative information that is updated with every new access, and underlie the execution and imagery of the action (Land et al., 2013; Schack et al., 2014). This suggests that involving two or more modalities in the learning process might result in a richer representation that involves more complementary information and therefore leads to a better learning outcome. In support of this view, Rosenblum et al. (2017) propose that the architecture of the brain implies perceptual parity between the senses, in particular between audition and vision, and that cross-sensory integration occurs completely and early in the perceptual stream. In the current study, the second learning step in fact consisted of more than just presentation of the complementary mode of information. In addition to the additional observation or listening to the verbal instruction, the participants had already practiced and performed the movement phrase several times. The second step thereby contained more physical practice and performance in addition to the additional information. It can thereby not be concluded that the development from Step 1 to Step 2 was entirely due to richer information. Adding another condition with the same modality in Step 1 and Step 2 would have helped to clarify this issue.

After the first learning step and in the retention test, the phrase initially leaned from visual observation was reproduced more completely than the phrase initially learned from verbal instruction. Crucially, the phrase initially learned from verbal instruction was reproduced less completely in the retention test than after the second learning step, whereas no such difference was found for the phrase initially leaned from visual observation. These results clearly indicate that initial learning from observation (complemented later by verbal information) was more successful in terms of recall and reproduction of the learnt material than initial learning from verbal instruction (complemented later by visual information). The superiority of visual observation as initial source of information is in line with previous findings supporting the view that learning from observation is the major learning mode for motor actions and is most successful in terms of the time spent learning and accuracy of the outcome (e.g., Schmidt, 1975, 2003; Schmidt and Lee, 1998; Hodges et al., 2007). Observational learning is considered to be mediated through the activation of shared neural correlates of action execution, observation and simulation (Jeannerod, 1995, 2004) as well as through the involvement of visual pathways for action perception in working memory processes (Vicary and Stevens, 2014; Vicary et al., 2014). The finding that AMo ratings were better for the first part of each phrase than for the middle and last part corresponds to the primacy effect found in the completeness scores; in both cases, the effect was more prominent in the Verbal-first than in the Visual-first condition. These findings support previous results according to which primacy effects, but no recency effects were found with regards to the learning of action sequences (Allard and Starkes, 1991; Wachowicz et al., 2011).

Corroborating the results for the completeness scores, expert ratings for AMo were generally better for the Visual-first condition than for the Verbal-fist condition after both learning steps. In the Verbal-first condition, expert ratings for AMo were better after the second than after the first learning step, whereas no difference between learning steps was found in the Visual-first condition. According to the dance experts' evaluation, approximation of the model phrase was generally better after visual learning than after learning from verbal instruction. In contrast, expert rating for the participants' individual interpretation of the learned movement phrases did not improve from learning Step 1 to Step 2 in either condition, and only for a subset of the questions, Visual-first ratings were slightly better than Verbal-first ratings after Step 1. The individual movement interpretation was obviously less sensitive to the learning mode than model approximation and not depending on the availability of complementary information, showing that participants' ability to dance and interpret the phrases did not depend on the information they had received, and that it therefore was more than plain reproduction of the movement, but rather a personal creative process.

In contrast to our practice-based expectations, expert ratings for individual interpretation were not better for the Verbal-first condition than for the Visual-first condition. Additionally, according to the questionnaires, students did not show a general preference or stronger feeling of ownership for the verbally learned compared to the visually learned movement, but, in contrast, expressed a general preference (more enjoyable, more useful) and higher familiarity for learning dance movement from observation. With regards to the experimental task, participants perceived the visual information as clearer than the verbal information, and they liked the phrase learned in the Visual-first condition better and felt more confident dancing it. Generally, liking a phrase was linked to finding it easy to remember and to dance, and feeling confident was linked to recall performance. Together with the finding that students in general preferred dance learning from observation and found it more familiar, and the lack of effects of learning type (according to the learning type questionnaire), these results suggest that learning preference and performance might strongly depend on habit, or being used to learning dance movement in a specific way. Taking the dance pedagogue's observations into account according to which learning dance movement in absence of a visual model might lead to a stronger identification and better interpretation of the movement, it might be the case that the stressful situation of the experimental learning task (an unfamiliar lab setting, restricted time, the teachers and other people watching) might have worked against the less familiar and therefore potentially more cognitively demanding way of leaning dance movement without a visual model. Indeed, most students expressed after the experimental task that they considered the situation as slightly stressful and perceived some kind of stage fright, in particular because their teachers were watching them during the learning task. It can be speculated that in a more relaxed situation with more time and less pressure, learning dance movement from verbal instruction might have been a more rewarding experience for the students. In dance training, experimenting with learning and teaching modes and approaches to movement learning, including the variation of available sensory information, can be a promising means to break habits, broaden the students' spectrum of experiences and induce creative processes. Another aspect that might play a role here is the type of verbal material used. In the present study, we used verbal instructions that mainly described the movement (e.g., “Extend your left leg forward and your two arms sideways to the horizontal. Allow your right hand to continue moving until it arrives to a high diagonal.”), and only very few instances of metaphorical language or images (“Feel the wind from the back that shifts your weight forward; let your upper body respond.”). There is some evidence that metaphorical language might work better in dance-related contexts than pure movement description (e.g., Sawada et al., 2002). In this study, no clear distinction was made between descriptive and metaphorical language, however, it might be promising to compare the use of both types of language in a similar learning scenario.

Taken together, the results of the study support our first hypothesis, as well as findings from previous research on observational learning of movement, showing clearly that initial observation of a human model is superior with respect to the recall and reproduction of the movement compared to initial learning movement from verbal instruction or movement description. It has to be pointed out that due to the design of this study we have not tested pure learning from observation to pure learning from acoustically presented verbal instruction, but two different approaches to learning a dance phrase based on either visual observation or verbal instruction as initial mode, later on followed by complementary verbal or visual information, respectively. Therefore, conclusions about learning from on or the other source of information exclusively can only be drawn with regards to the completeness scores and experts' ratings after Step 1, but not after Step 2 or the retention test. Accordingly, with regards to the retention test, we cannot draw any conclusion about the comparison of learning from visual vs. verbal information exclusively, but we compare the two modes as initial source of information.

In contrast to our expectations that were based on experience from dance training, initial learning from verbal instruction in absence of a visual model (that was presented only in the later learning step) did neither result in better ratings for the individual interpretation in movement performance, nor in a stronger personal preference for, or identification with, the learnt material, leading to the rejection of our second hypothesis. The third hypothesis, namely that performance after the second learning step would be improved due to the combination of different modes of presentation and an increased amount of practice, was supported by the completeness scores for both conditions, whereas the expert ratings of approximation of the model, but not for individual interpretation supported this hypothesis for the material initially learned through verbal instruction. These findings again support the superiority of visual observation as initial learning mode with regard to the approximation of the model phrase.

To round up the discussion of the presented results, it has to be admitted that the findings are potentially limited by the rather small number of 18 participants, as is often the case with participants who are experts in particular fields of practice, such as dance or sports (this issue was increased further by the unforeseen and unfortunate reduction to *N* = 13 at the time of the retention test). Due to this limitation, a within-groups design was chosen two comparable dance phrases, with each participant learning one phrase in one condition and the other one in the other condition. A between-groups design (with only one to-be-learnt dance phrase) would have resulted in a cleaner design, but would have required a larger group of participants. On the other hand, a clear (and rare) advantage of the available group of participants was that as they were all studying dance together in the same class, with the same teachers, and therefore were as similar in their dance experience as it might be possible in the real world. Another way of keeping the design simpler and thus easier to interpret would have been to leave out the second learning step and to concentrate on the effects of purely observational vs. verbal learning. This would have made the retention test easier to interpret and potentially more meaningful (in particular with the full number of participants). Movement learning in dance is hardly ever based on one learning mode alone, but is commonly supported by a combination of visual observation, verbal instructions and feedback, and kinesthetic information achieved through physical movement, typically applied at the same time. In the present study, our aim was to disentangle visual and verbal information about a to-be-learnt dance phrase without removing too much of the “normal” dance learning context. A simpler design that separates visual and verbal learning throughout the study and controls more rigorously for the participants' actions during the learning task would have made the experimental conditions clearer for interpretation, in particular at the time of retention.

## Conclusions

In general, our results corroborate the superiority of visual observation of a human model as means for learning complex movement sequences in dance. More precisely, visual observation as initial learning mode (followed by verbal instruction) was found to result in better outcomes than verbal instruction as initial learning mode (followed by observation of a visual model). Given the findings of this study and arguments brought forward in the literature in support observational learning (e.g., Vicary and Stevens, 2014; Vicary et al., 2014), it might seem surprising that the participants were well able to learn the complex movement sequences exclusively from verbal information. Even though the participants of this study were students still at the beginning of their professional dance education, they clearly demonstrated remarkable abilities in movement learning that can be considered a specific feature of dance expertise. Dancers' specific learning and memory skills have been addressed in several studies (e.g., Stevens and McKechnie, 2005; Bläsing et al., 2009; Wachowicz et al., 2011; Bläsing and Schack, 2012), and growing evidence exists that dance experts differ from non-dancers not only with regards to quantitative aspects, such as working memory capacity, but also qualitative ones, including specific modes and strategies of storage and retrieval, as well as the interaction between memory processing and motor performance (see Sevdalis and Keller, 2011; Bläsing et al., 2012; Stevens, 2017). Furthermore, dancers' enhanced skills in motor imagery have been found to contribute substantially to their learning and performance skills by increasing the efficiency of kinaesthetic sensations and making images more complex and vivid (e.g., Nordin and Cumming, 2007; Golomer et al., 2008; Fink et al., 2009), and might thus have supported the students' performance in learning movement from verbal instruction in the present study. Additionally, dancers acquire specific strategies and techniques to support movement learning and recall, such as marking dance movements by hand gestures or reduced full-body movements (Kirsh, 2011; Warburton et al., 2013). Marking can be considered a cognitive tool that makes use of the same cognitive functions as executing, observing and mentally simulating motor actions (Jeannerod, 1995, 2004) and that can thereby serve as a kind of (partly) externalized memory (Allard and Starkes, 1991; Stevens, 2017) or as cognitive offline-strategy in which the body is used to reduce cognitive load (Wilson, 2002). With regards to the current study, it can be reported as qualitative observation that students used a lot of different marking while watching or listening during the experimental learning tasks. Analysing the individual learning strategies (including marking techniques) applied by the participants of this study could be a promising next step to increase or understanding of movement learning in dance.

## Author contributions

BB, JC, JB, and TS have planned the study together. BB, JC, and JB have conducted the study and collected the data. BB has analyzed the data and written the manuscript. TS have provided input to the manuscript and feedback to several previous versions.

### Conflict of interest statement

The authors declare that the research was conducted in the absence of any commercial or financial relationships that could be construed as a potential conflict of interest.
