# Supplementary material for: Watching or Listening: How Visual and Verbal Information Contribute to Learning a Complex Dance Phrase
Source: Front Psychol. 2018 Nov 30;9:2371. doi: 10.3389/fpsyg.2018.02371 (PMC6284028; doi:10.3389/fpsyg.2018.02371)
Supplement: Supplementary file 3 [file Data_Sheet_1.PDF]

Dance Phrase 1

Part 1

1. Stand facing the front left diagonal of the room in first position. At the same time extend your left leg forward and your two arms sideways to the horizontal. Allow your right hand to continue moving until it arrives to a high diagonal.

2. Gradually let the shape melt back into its beginning position as you shift your weight into the right hip, bending both knees, sinking your head to the left to make a big C-curve.

3. Continue into falling, then catch the weight with a step of the left leg crossing to the right. Follow with two steps sideward, in the same direction while throwing both arms in front of your shoulders.

4. Keeping your arms close to you, spiral to the right diagonal, then, kick your right leg, left arm and head forward as you throw your right arm behind you.

5. Bring the energy back into you quickly bending both elbows and the right knee close to the body, spine vertical.

Part 2

6. Drop your arms and take a step back onto your right leg turning fully around while dragging your left leg behind you. Finish with the weight low, left leg behind, spine rounded forward, arms wrapped around the body, right arm front, left arm back.

7. Stretch your legs and gradually lengthen your spine horizontally. Allow your arms to follow the succession of your spine, right front, left back.

Part 3

8. Shift and bend into your right knee. Swing your left arm forward and up as you slide your left foot to your standing leg.

9. Rotate and open your whole left side, looking into the palm of your left hand.

10. Keeping your left arm over your head, take six steps forward in a circular pathway to the left and end facing the left side of the room closing in a parallel position.

11. Let your left arm sink downward in an easy curve in front of you as you send the right fingertips upwards passing in front of your face. Continue to rise upwards and as you reach the full extension of your body suspend.
