# Supplementary material for: Watching or Listening: How Visual and Verbal Information Contribute to Learning a Complex Dance Phrase
Source: Front Psychol. 2018 Nov 30;9:2371. doi: 10.3389/fpsyg.2018.02371 (PMC6284028; doi:10.3389/fpsyg.2018.02371)
Supplement: Supplementary file 4 [file Data_Sheet_2.PDF]

Dance Phrase 2

Part 1

1. Stand facing the left front diagonal of the room in parallel position. Feel the wind from the back that shifts your weight forward; let your upper body respond. Allow your body to move back and take the impulse again to move forward, allowing your weight to transfer from your heels to your toes.

2. Once again shift back, this time falling onto your left leg, and follow with another step back, long and grounded, ending in a low lunge position, torso diagonal.

3. Staying low, kick your right leg forward and your arms outwards to the sides as you twist your torso in opposition to the kick.

4. Quickly bend your leg and arms into your center with a half turn to the right.

Part 2

5. Let the weight of your arms and center sink down on your left leg as your torso melts in a side-bend to the left and your leg extends sideways in opposition.

6. Shift your weight onto the extended leg while your left arm describes a horizontal surface in front of you, reaching your torso over to the right side and bringing your left foot to the knee.

7. While maintaining this opposition, fall, and leading with the right arm as if embracing a partner, take three steps in a semicircle to the left ending facing the forward right diagonal.

8. Transfer your weight sideways onto a bent right leg, spine curved forward, arms hanging.

9. Gradually lengthen your spine from head to tail in the horizontal, allowing your left arm to open sideways as part of this movement.

Part 3

10. Let your arm drop like a pendulum and use the returning impulse to carry your body over to the left leg, feeling the diagonal opposition between left arm high and right leg low.

11. Letting your left arm sink, reach with your right leg behind you and take two steps all the way around to face front. Continue with a third step to close in a parallel position, knees bent, spine curved forward.
