# Supplementary material for: Watching or Listening: How Visual and Verbal Information Contribute to Learning a Complex Dance Phrase
Source: Front Psychol. 2018 Nov 30;9:2371. doi: 10.3389/fpsyg.2018.02371 (PMC6284028; doi:10.3389/fpsyg.2018.02371)
Supplement: Supplementary file 5 [file Data_Sheet_3.PDF]

Table A: Comparison of mean completeness scores for the individual elements of each dance phrase.

	1	2	3	4	5	6	7	8	9	10	11
1						-2.193<br>.028	-2.429<br>.015		-1.990<br>.047		
2				-2.549<br>.011	-2.038<br>.042	-2.371<br>.018	-2.666<br>.008			-2.108<br>.035	
3	-2.028<br>.043			-1.990<br>.047						-2.176<br>.030	
4	-2.437<br>.015										
5	-2.301<br>.021										
6	-2.827<br>005										
7	-2.941<br>.003	-2.402<br>.016		-2.091<br>.037							
8	-2.810<br>.005	-1.994<br>.046									
9	-2.941<br>.003	-2.403<br>.016	-2.406<br>.016	-2.452<br>.014							
10	-2.499<br>.012								-2.132<br>.033		
11	-2,589<br>,010						-2,182<br>,029		-2,559<br>,010		

Light grey: Visual-first condition; dark grey: Verbal-first condition. In the cells, Z and p values for significant results (Wilcoxon signed-rank tests) are displayed, empty cells represent non-significant results.
