# Supplementary material for: Watching or Listening: How Visual and Verbal Information Contribute to Learning a Complex Dance Phrase
Source: Front Psychol. 2018 Nov 30;9:2371. doi: 10.3389/fpsyg.2018.02371 (PMC6284028; doi:10.3389/fpsyg.2018.02371)
Supplement: Supplementary file 6 [file Data_Sheet_4.PDF]

Table B: Results of Wilcoxon signed-rank tests comparing expert ratings.

		Verbal-first	Visual-first	Step 1	Step 2
**AMo**					
A1.1	Z	**-2.294**	-0.036	**-1.936**	***-1.889***
	p	**.022**	.971	***.053***	***.059***
A1.2	Z	**-3.570**	-1.647	**-3.401**	**-3.216**
	p	**.000**	.100	**.001**	**.001**
A1.3	Z	**-2.491**	-0.792	**-2.663**	**-2.790**
	p	**.013**	.428	**.008**	**.005**
A1.4	Z	**-3.074**	-1.084	**-2.938**	**-2.498**
	p	**.002**	.279	**.003**	**.012**
A1.5	Z	**-2.235**	-0.700	**-2.951**	**-2.563**
	p	**.025**	.484	**.003**	**.010**
A1.6	Z	**-3.346**	-0.397	**-2.847**	-1.366
	p	**.001**	.691	**.004**	.172
A1	Z	**-3.625**	-0.403	**-3.245**	**-2.593**
	p	**.000**	.687	**.001**	**.010**
A2.1	Z	**-3.488**	-1.026	**-3.403**	**-2.899**
	p	**.000**	.305	**.001**	**.004**
A2.2	Z	**-3.142**	-1.489	**-3.093**	**-3.136**
	p	**.002**	.136	**.002**	**.002**
A2.3	Z	**-3.351**	-0.499	**-3.331**	**-2.143**
	p	**.001**	.618	**.001**	**.032**
A2	Z	**-3.686**	-1.303	**-3.519**	**-3.133**
	p	**.000**	.193	**.000**	**.002**
**IndI**					
B1.1	Z	-0.905	-0.412	-0.862	-1.428
	p	.366	.680	.389	.153
B1.2	Z	-0.540	-0.165	**-1.999**	***-1.934***
	p	.589	.869	**.046**	***.053***
B1.3	Z	-0.928	-1.095	**-2.167**	-0.612
	p	.353	.273	**.030**	.541
B1.4	Z	-1.447	-0.275	**-2.127**	-0.553
	p	.148	.783	**.033**	.580
B1.5	Z	-1.125	-0.816	-1.693	-0.728
	p	.261	.414	.090	.467
B1	Z	-1.142	-0.095	-1.786	-1.309
	p	.254	.924	.074	.190
B2	Z	-1.387	-1.667	-1.705	***-1.941***
	p	.166	.096	.088	***.052***

AMo: Approximation of the model; IndI: individual Interpretation; significant results are written in bold letters, tendencies are written in bold italics letters, non-significant results are written in grey letters; cells with categorial ratings A1 (A1.1-6), A2 (A2.1-3) and B1 (B1.1-5) are marked in grey.
